# Ruxolitinib Ameliorates Airway Hyperresponsiveness and Lung Inflammation in a Corticosteroid-Resistant Murine Model of Severe Asthma

**DOI:** 10.3389/fimmu.2021.786238

**Published:** 2021-10-29

**Authors:** Hariharan Subramanian, Tanwir Hashem, Devika Bahal, Ananth K. Kammala, Kanedra Thaxton, Rupali Das

**Affiliations:** ^1^ Department of Physiology, College of Human Medicine, Michigan State University, East Lansing, MI, United States; ^2^ College of Natural Science, Michigan State University, East Lansing, MI, United States; ^3^ College of Veterinary Medicine, Michigan State University, East Lansing, MI, United States

**Keywords:** severe asthma, corticosteroid resistance, T2-low asthma, ruxolitinib, house dust mite extract (HDME), airway hyperresponsiveness (AHR), lung inflammation, interleukin (IL)-17

## Abstract

Asthma prevalence has increased considerably over the decades and it is now considered as one of the most common chronic disorders in the world. While the current anti-asthmatic therapies are effective for most asthma patients, there are 5-10% subjects whose disease is not controlled by such agents and they account for about 50% of the asthma-associated healthcare costs. Such patients develop severe asthma (SA), a condition characterized by a dominant Th1/Th17 cytokine response that is accompanied by Type 2 (T2)-low endotype. As JAK (Janus Kinase) signaling is very important for the activation of several cytokine pathways, we examined whether inhibition of JAKs might lessen the clinical and laboratory manifestations of SA. To that end, we employed a recently described murine model that recapitulates the complex immune response identified in the airways of human SA patients. To induce SA, mice were sensitized with house dust mite extract (HDME) and cyclic (c)-di-GMP and then subsequently challenged with HDME and a lower dose of c-di-GMP. In this model, treatment with the JAK inhibitor, Ruxolitinib, significantly ameliorated all the features of SA, including airway hyperresponsiveness and lung inflammation as well as total IgE antibody titers. Thus, these studies highlight JAKs as critical targets for mitigating the hyper-inflammation that occurs in SA and provide the framework for their incorporation into future clinical trials for patients that have severe or difficult-to manage asthma.

## Introduction

Asthma affects more than 300 million people worldwide and is associated with significant morbidity and mortality (1, 2). It is deemed as a major public health issue as the direct medical expense of asthma treatment and the indirect costs associated with time lost from work and premature death contribute to the increased global economic burden (2). Clinically, asthma is a chronic inflammatory disease often characterized by recurrent episodes of wheezing, coughing, shortness of breath and chest tightness that is caused due to airway hyperresponsiveness (AHR), bronchoconstriction, increased mucus production and airway remodeling (1, 2).

**Figure 1:**
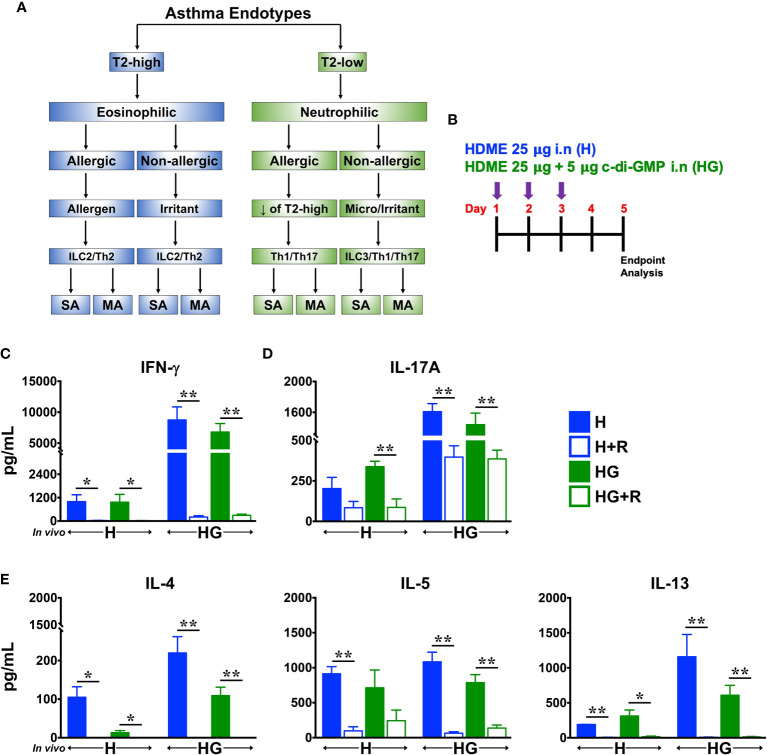
Ruxolitinib reduces HDME- and HDME+c-di-GMP-induced Th1, Th2/T2 and Th17 cytokine production *in vitro*. **(A)** Classification of asthma endotypes. **(B)** Schematics of short-term *in vivo* sensitization. Balb/c mice were challenged intranasally (i.n) with HDME (25 μg) or HDME (25 μg)+c-di-GMP (5 μg) for 3 consecutive days. Forty-eight hours after the last injection, mice were sacrificed and lungs were harvested for cytokine analysis. **(C–E)** Lung mononuclear cells were isolated and cultured *ex vivo* in the presence of either HDME (H) or HMDE+c-di-GMP (HG), with or without Ruxolitinib (R), as indicated. After 72 hours, cell culture supernatants were analyzed for **(C)** IFN-γ, **(D)** IL-17A, and **(E)** T2 cytokines (IL-4, IL-5 and IL-13). Data is shown as mean ± SEM and pooled from 3 independent experiments with a total of 3–9 mice per cohort. Statistical significance was determined by Student’s unpaired *t* test with Welch’s correction. **p ≤ 0.01, *p ≤ 0.05. SA, severe asthma; MA, mild asthma; micro, microbes.

Traditionally, asthma is defined as two distinct forms: non-allergic and allergic phenotypes, the latter being the most common form of asthma and is caused by sensitization to allergens such as pollen, mold, cockroach, pet dander, and dust mites (1). However, it is now well accepted that asthma is a complex heterogenous disease that is regulated by various inflammatory pathways (1, 2). Thus, based on the pathophysiologic mechanisms, asthma is now classified as Type 2 (T2)-high and T2-low endotypes, as depicted in **Figure 1A**. In T2-high asthma, CD4+T helper 2 (Th2) cells and innate lymphoid cells group 2 (ILC2), eosinophils, immunoglobulin E (IgE) and T2 cytokines such as interleukin (IL)-4, IL-5 and IL-13 contribute to its pathogenesis. In contrast, the pathobiology of T2-low asthma typically involves Th1 and Th17 cells, neutrophils and several proinflammatory cytokines such as IL-1β, IL-6, IL-17A, IL-17F, IFN-γ and TNF-α (2).

The current anti-inflammatory drugs for asthma treatment include corticosteroids (CS) and anti-leukotrienes, that are effective for most of asthma patients (2). However, there are about 5-10% subjects that develop severe asthma (SA) and do not respond to these agents (1). Such severe asthmatics require frequent hospitalizations and/or need emergency care, contributing up to 50% of health costs associated with asthma (1). In a prior study (3), Raundhal et al. reported that SA patients have a dominant Th1 immune response inspite of ongoing treatment with high doses of CS, highlighting the need for the development of newer and effective therapies.

Most pro-inflammatory cytokines signal through Janus Kinase (JAK) proteins (4). JAKs are a family of four tyrosine kinases (JAK1, JAK2, JAK3 and Tyk2) that selectively associate with cytokine receptor chains and mediate signaling by phosphorylating tyrosine residues on themselves, the cytokine receptor chains and STAT (signal transducer and activator of transcription) proteins (4). JAK1 plays a major role in the signaling of several proinflammatory cytokines, often in association with other JAK family members, such as JAK2 or JAK3 (4, 5). A number of JAK inhibitors have been developed for clinical use in inflammatory diseases, including asthma (5, 6). However, the effect of JAK inhibitors on the immunopathology of CS-resistant SA remains to be investigated.

In the current study, we examined the effect of Ruxolitinib (7), a potent inhibitor of JAK1/2, on the pathogenesis of CS-resistant SA. To that end, we used a recently developed murine model of SA that recapitulates the immune pathophysiology of severe asthmatics unresponsive to CS (3). Consistent with a prior report (3), we observed that intranasal administration of HDME and c-di-GMP (a mucosal adjuvant as well as a potent STING [Stimulator of Interferon genes] agonist) induced AHR and lung inflammation in mice. These SA mice exhibited high serum IgE levels and had significantly increased numbers of both eosinophils and neutrophils in their lungs. Consistent with the mixed granulocytic infiltration, lungs of SA mice expressed enhanced gene transcripts of chemokines and cytokines that drive eosinophilic and neutrophilic inflammation. Importantly, Ruxolitinib significantly reduced HDME+c-di-GMP-mediated AHR, lung inflammation and serum IgE levels. However, this amelioration in the SA features was associated with suppression of cytokines and chemokines that predominantly regulate Th1 and T2 immune response, independent of the cellular factors that regulate neutrophil recruitment and function. Lastly, we demonstrate that Ruxolitinib critically modulates expression of several microRNAs that have known roles in the pathogenesis of SA.

## Materials And Methods

### Mice

Balb/c mice were purchased from Jackson Laboratories (Bar Harbor, ME); bred and housed under specific pathogen-free conditions. Female mice (8-10 weeks old) were used for experiments. All animal studies were approved by the Institutional Animal Care and Use Committee at the Michigan State University (protocol number: PROTO202000162).

### Mouse Model of Severe Asthma

**Figure 2:**
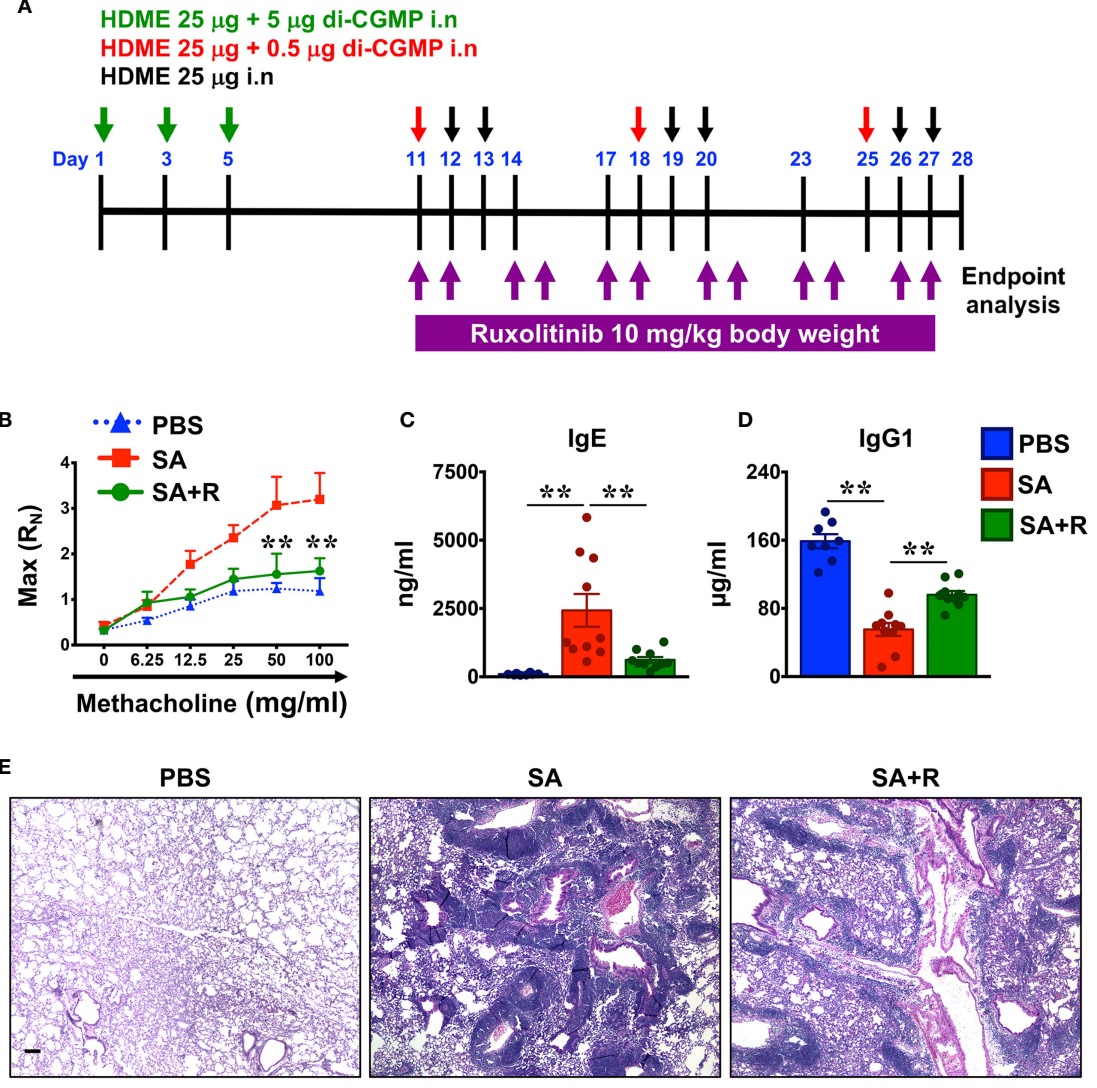
Ruxolitinib ameliorates AHR as well as reduces IgE production and lung inflammation in a mouse model of SA. **(A)** Schematics of the severe asthma (SA) model used in the study. Mice were sensitized with HDME and c-di-GMP (green arrows) and subsequently challenged with HDME and a lower dose of c-di-GMP (red arrows) or HDME alone (black arrors) as indicated. During the challenge phase, mice were also treated intranasally (i.n) with Ruxolitinib (R, 10 mg/kg body weight) for two consecutive days followed by a day of rest for a total of 12 injections. **(B)** Twenty-four hours after the last injection, mice were analyzed for AHR. Assessment of resistance to airflow in central airways (Newtonian resistance [Rn]) after challenge with increasing doses of methacholine (Mch). Data is shown as mean ± SEM and pooled from 3 independent experiments with a total of 9-12 mice per cohort. Statistical significance was determined by two-way ANOVA. **p ≤ 0.01. **(C, D)** Levels of serum IgE **(C)** and IgG1 **(D)** in PBS-treated, SA (challenged with HMDE+c-di-GMP) or SA mice treated with Ruxolitinib (SA+R). Data is shown as mean ± SEM and pooled from 3 independent experiments with a total of 8-13 mice in each group. Statistical significance was determined by Student’s unpaired *t* test with Welch’s correction. **p ≤ 0.01. **(E)** Hematoxylin and eosin (H&E) staining of lung section of PBS-treated, SA and SA+R mice. Scale bar: 100μm. Representative micrographs of the lung sections at 4X magnification. Data is representative of 3 independent experiments with a total of 9 mice per cohort.

A previously described mouse model of severe asthma was used (3). Age-matched Balb/c female mice were intranasally (i.n) sensitized using 25μg of house dust mite extract (HDME) (1μg/μL, 28750 EU/vial; Stallergenes Greer, UK) mixed with 5μg of c-di-GMP (Invivogen, San Diego, CA) on days 1, 3 and 5. After 5 days of rest, mice were re-challenged with HDME (25μg) and a lower dose of c-di-GMP (0.5μg) on day 11 and with HDME only (25μg) on days 12 and 13. Mice were similarly re-challenged two more times on days 18-20 and 25-27. For all i.n administrations, mice were anasthesized with 2.5% isofluorane. HDME was reconstituted in sterile PBS at a concentration of 2μg of protein/μL. The amount of HDME protein, Derp1 and endotoxin was 50 μg, 3.1μg and 38.4 EU respectively per injection in a volume of 25μL. Ruxolitinib (LC Laboratories, Woburn, MA) was prepared in citrate buffer (pH 3.5) with 20% (wt/vol) Captisol (Ligand Pharmaceuticals, La Jolla, CA) and was given i.n (10 mg/kg body weight) starting on the first day of the challenge for 2 consecutive days followed by a day of rest for a total of 12 injections. Schematic of this model is shown in **Figure 2A**. Twenty-four hours after the final HDME challenge, mice were anesthetized for measurement of airway hyperresponsiveness (AHR) and then sacrificed for collection of blood, bronchoalveolar lavage fluid (BALF) and lung tissue for various endpoint analysis.

### AHR Assessment

Mice were anesthetized using 100 mg/kg ketamine (Henry Schein Animal Health, Dublin, OH), 10 mg/kg xylazine (Akorn, Lake Forest, IL) and 3 mg/kg acepromazine (Henry Schein Animal Health, Dublin, OH) through intraperitoneal (i.p) injection. Subsequently mice were tracheostomized and airway mechanics was measured using flexiVent (SCIREQ^®^, Quebec, Canada). Assessment of AHR such as central airway resistance [Newtonian resistance (Rn)] was measured by a methacholine (MCh; Sigma-Aldrich, St. Louis, MO) challenge test with increasing doses of MCh (0-100 mg/ml, as indicated in **Figure 2B**) in 100 μL volume, using a nebulizer.

### Serum and Bronchoalveolar Lavage Fluid (BALF) Collection

Blood was collected from the superior mesenteric vein of the mouse and left at 4°C overnight. Serum was collected the next day and analyzed for total IgE and IgG1 using commercially available ELISA kits from Invitrogen (Carlsbad, CA). For BALF collection, mice were euthanized and whole lung was lavaged with 0.6 ml of sterile PBS three times. The resultant BALF was centrifuged to separate the cellular components from the supernatants. Total BALF cellularity was determined using a hemacytometer.

### Lung Histology

The lungs were infused *via* the trachea with 10% buffered formalin. After excision, the lungs were immersed in fresh 10% formalin overnight. Samples were then embedded in paraffin, cut into 5-μm-thick sections and stained with hematoxylin and eosin (H&E). Digital images of sections were obtained using a Nikon Eclipse 50i microscope (Nikon, Japan) equipped with a INFINITY-3 digital color camera (Lumenera Corporation, Canada), and INFINITY ANALYZE 6.5.4 software.

### Quantitative Real-Time PCR

Lungs were dissociated in TRIzol solution (Thermo Fisher Scientific, Waltham, MA) using a high-speed homogenizer (Fisher Scientific, Hampton, NH) and total RNA was extracted as per manufacturer’s protocol. RNA (2 μg) was reverse transcribed into cDNA using SuperScript III in a 20 μl reaction volume or using the Taqman Advanced miRNA cDNA synthesis kit (for microRNA analysis) according to the manufacturer’s instructions (Applied Biosystems, Foster City, CA). Real-time quantitative PCR was performed using Quant Studio™ 3 system (Applied Biosystems) with validated Taqman primers and Fast Advanced Master Mix according to manufacturer’s instructions. Relative gene expression data (fold change) between samples was accomplished using the 2^-ΔΔCt^ method. GAPDH (for gene expression) or 18S (for miRNA analysis) was used as the internal reference control.

### Isolation of Immune Cell Populations From the Lungs

Lung samples were digested with collagenase P (1 mg/ml, Roche Diagnostics, Indianapolis, IN) at 37°C for 30 minutes. Single cell suspension was obtained by passing the digested tissue through a 70 μm cell strainer (Alkali Scientific Inc, Fort Lauderdale, FL) with a plunger. Lung immune cells were then isolated using density centrifugation with Percoll (GE, Piscataway, NJ). The isolated cells were washed and resuspended in RPMI media (Life Technologies, Carlsbad, CA) conditioned with 10% FBS (Atlanta Biologicals, Flowery Branch, GA) and 1% penicillin-streptomycin (Mediatech Inc, Manassas,VA). Total lung cellularity was determined using a hemacytometer.

### Re-Stimulation of Lung Immune Cells *In Vitro*

Lung immune cells (0.2 x 10 (6) cells/well) were plated with either HDME (3μg/well) or HDME (3μg/well) + c-di-GMP (0.5 μg/well) without or with Ruxolitinib (1μM/well) in triplicates and incubated at 37°C. After 72 hours, culture supernatant was collected and levels of IFN-γ, IL-17A, IL-4, IL-5 and IL-13 were measured using ELISA kits (Invitrogen and BD Bioscience).

### Antibodies and Flow Cytometry

The antibodies used for immunofluorescence staining include CD4, CD11b, B220, DX5, TCRβ, and SiglecF (BD Biosciences). CD8a, CD11c, IA/IE (MHCII), and Ly6G were from Biolegend, San Diego, CA. Fluorochrome conjugated CD1d-tetramer (CD1d-Tet) loaded with glycolipid antigen (PBS57), or unloaded controls were provided by the NIH Tetramer Core Facility (Emory University, Atlanta, GA). Data was collected on a LSRII flow cytometer (BD Biosciences) and analyzed using FlowJo software (FlowJo LLC, Ashland, OR).

### Statistics

Statistical significance was determined using GraphPad Prism software (GraphPad, San Diego, CA). Student’s *t*-test with Welch’s correction or two-way ANOVA with Tukey’s multiple comparisons test were used as indicated in the figure legends. Significance is shown as *(p<0.05), **(p<0.01), or ns for non-significant values.

## Results

### Ruxolitinib Suppresses HDME- and HDME+c-di-GMP-Induced Cytokine Production *In Vitro*

Type 2 (T2)-high asthma is associated with elevated levels of Th2 cytokines such as IL-4, IL-5 and IL-13 whereas T2-low asthma is mainly linked to the activation of Th1 and Th17 cells (2) (**Figure 1A**). Others (8) and we (9) have previously shown that repeated intransal exposure to HDME (a naturally inhaled antigen) results in a T2-polarized eosinophilic lung inflammation and airway hyperresponsiveness (AHR) in mice, similar to that seen in human asthmatics. In contrast, the second messenger c-di-GMP induces a robust Th1/Th17 but a modest T2 response (3). To investigate the effect of Ruxolitinib on allergen-induced cytokine production, we sensitized wild-type Balb/c mice with repeated high doses of HDME (H) or HDME+c-di-GMP (HG) (**Figure 1B**). We observed that lung immune cells from mice sensitized with HG produced significantly higher levels of IFN-γ and IL-17A (hallmark cytokines of Th1 and Th17 cells, respectively) when stimulated with either HDME or HG *in vitro*, as compared to HDME-treated mice (**Figures 1C, D**). However, comparable amounts of T2 cytokines (with the exception of IL-13) were produced by lung cells from mice sensitized with either HDME or HG (**Figure 1E**). Strikingly, when lung immune cells from these animals were stimulated with either HDME or HG in presence of Ruxolitinib *in vitro*; levels of all the cytokines in culture supernatants were significantly reduced (**Figures 1C** **–E**).

### Ruxolitinib Attenuates AHR, IgE Production and Lung Inflammation in SA Mice

In light of the *in vitro* findings (**Figure 1**), we next determined the effect of Ruxolitinib *in vivo*, using a murine model that recapitulates the corticosteroid (CS)-refractory immune response seen in severe human asthmatics (**Figure 2A**). In this model (3), intranasal (i.n) injection of HG induces a mixed Th1 and Th17 immune response that is accompanied with a low T2 activity. As depicted in **Figure 2A**, we administered Ruxolitinib i.n in the challenge phase; once a day for two consecutive days. After a day of rest, the drug treatment was repeated until the end of the study for a total of 6 treatment sets (**Figure 2A**). PBS-control and severe asthma (SA: HG-treated) mice without or with Ruxolitinib (SA+R) were anesthetized and AHR [central airway resistance (Rn)] was assessed in response to methacholine (Mch) challenge. Compared to PBS controls, SA mice demonstrated significant elevation in Rn, in a dose-dependent manner over a range of 6.25–100 mg/ml of Mch. In contrast, SA+R mice manifested a dramatically decreased AHR, almost similar to levels observed in PBS-treated mice (**Figure 2B**).

Allergic asthma is characterized by elevated levels of serum IgE (10). Accordingly, we observed that serum IgE was significantly higher in SA mice as compared to the PBS controls (**Figure 2C**). IgE synthesis can occur through different biosynthetic pathways, either by “direct” class-switch recombination (CSR) from IgM, or through “sequential” switch from IgM to IgG1 and then from IgG1 to IgE (11). We observed that serum IgG1 levels were greatly reduced in SA mice (**Figure 2D**), suggesting that HG induces class-switch of IgG1 to IgE. Importantly, Ruxolitinib treatment significantly decreased serum IgE but increased IgG1 levels in SA+R mice (**Figures 2C, D**). Thus it is likely that Ruxolitinib regulates IgE levels *via* direct and/or sequential CSR events.

Acute exposure to allergen increases AHR that is often associated with airway inflammation characterized by an influx of inflammatory cells (10). Having observed that Ruxolitinib attenuates HG-induced AHR, we next determined the effect of Ruxolitinib on lung inflammation. Histological analysis of the lung sections revealed that while HG induced airway inflammation in SA mice, it was substantially attenuated in Ruxolitinib-treated animals. This reduction in lung inflammation was most evident around the peribronchial and perivascular areas of the lung tissue sections (**Figure 2E**).

### Decreased Lymphocytic Infiltration in the Lungs of Ruxolitinib-Treated SA Mice

**Figure 3:**
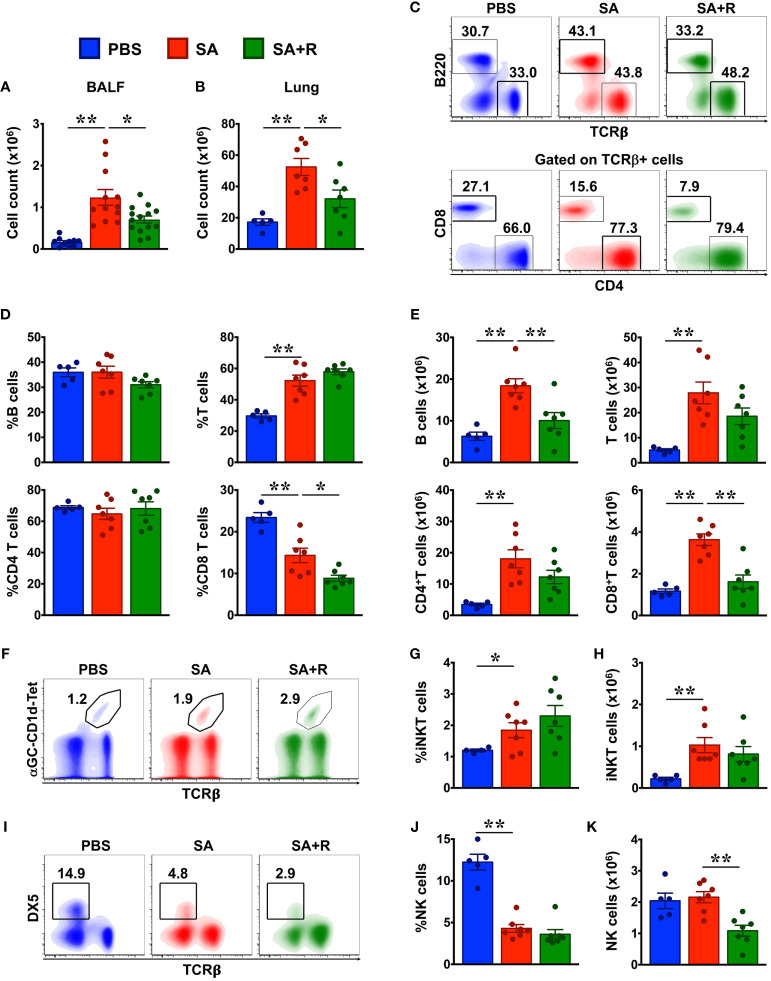
Reduced cellular infiltration in the BALF and lungs of SA mice treated with Ruxolitinib. **(A, B)** BALF **(A)** and lung tissues **(B)** from control (PBS-injected), SA and SA mice treated with Ruxolitinib (SA+R) were analyzed for total leukocyte counts. Data is shown as mean ± SEM and pooled from 3 independent experiments with a total of 5-13 (BALF) or 5-7 (lungs) mice per cohort. **(C–K)** Lung immune cells were analyzed for various lymphocytic populations by flow cytometry. Representative density plots **(C, F, I)** are from 2 independent experiments. Average frequency **(D, G, J)** and total cell numbers **(E, H, K)** of various lymphocytes from PBS, SA and SA+R mice were pooled. Data is presented as the mean of each group ± SEM of at least 5-7 mice per group. Statistical significance was determined using Student’s unpaired *t* test with Welch’s correction. *p < 0.05, **p < 0.01.

**Figure 4:**
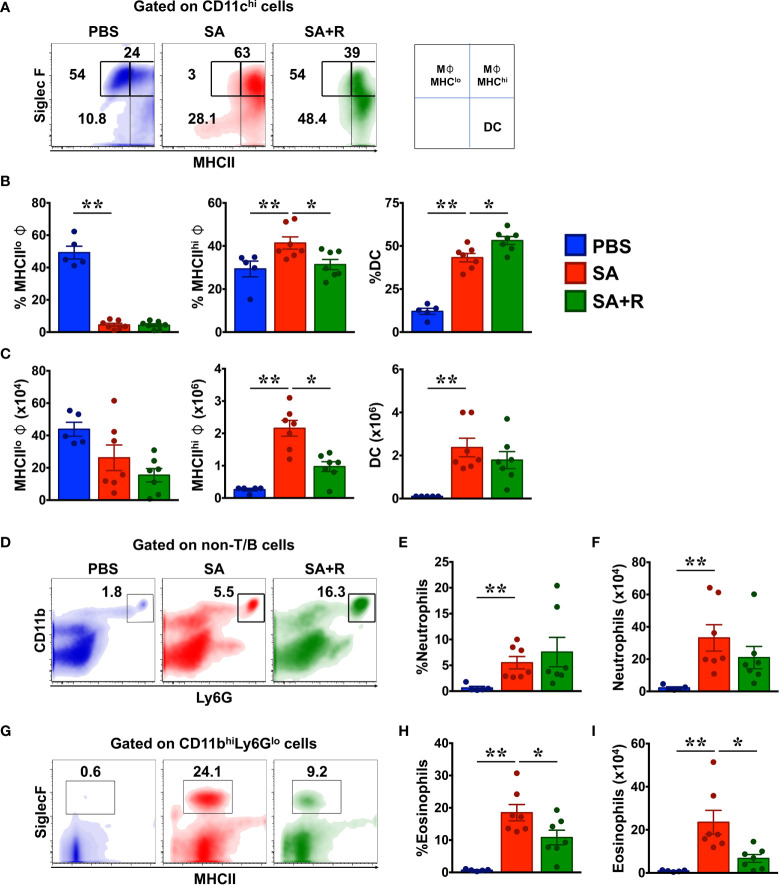
Ruxolitinib reduces M1 macrophages and eosinophils but not neutrophils in the lungs of SA mice. **(A–I)** Lung cells were analyzed for various innate immune cell populations by flow cytometry. Representative density plots **(A, D, G)**, average frequency **(B, E, H)** and total cell numbers **(C, F, I)** of macrophages, dendritic cells (DC), neutrophils and eosinophils from PBS, SA and SA+R mice are shown. Data is pooled from 2 independent experiments and presented as mean ±; SEM of at least 5-7 mice per group. Statistical significance was determined using Student’s unpaired *t* test with Welch’s correction.*p < 0.05, **p < 0.01.

To determine whether the decreased lung pathology observed in the SA+R mice was due to a reduction in the recruitment of inflammatory cells to the airways, we harvested BALF and lung tissue and analyzed for total cell counts. We observed that following i.n. challenge with HG, cellular infiltration in both the BALF and the lungs was significantly elevated. However, this was markedly reduced by Ruxolitinib (**Figures 3A, B**), which was consistent with the lung histology. To determine the relative makeup of the cellular inflammation, we used mutli-color flow cytometry and analyzed lung immune cells from PBS controls, SA and SA+R mice (**Figures 3C–K**, **4**). Increased B cell numbers are found in allergic lungs, as well as in the sputum of asthma patients (12, 13). Futhermore, while T lymphocytes, specifically CD4+T-cells play a major role in the pathophysiology of asthma, particularly eosinophilic and CS-responsive asthma, CD8+ T cells are important effector cells in the pathogenesis of severe CS-resistant asthma (14). Consistent with these reports, we observed that the cell numbers of all these lymphocytic populations were dramatically increased in the lungs of SA mice (**Figures 3C** **–E**). Invariant natural killer T (iNKT) cells are glycolipid-reactive innate T lymphocytes that play a critical role in the development of AHR (15). Accordingly, we observed that both the frequency and cell counts of iNKTs were increased in the lungs of SA mice (**Figures 3F** **–H**). In contrast, natural killer (NK) cell incidence was substantially reduced in these animals (**Figures 3I, J**). This was surprising as NK cells promote T cell-mediated acute allergic inflammation (16). Subsequently, when we evaluated these lymphocytic populations in SA+R mice, we observed that Ruxolitinib had the most dramatic impact on the frequency and number of CD8+ T cells (**Figures 3C–E**), with concomitant decrease in B and NK cell counts (**Figures 3E, K**).

### Treatment With Ruxolitinib Reduced Macrophages and Eosinophils but Not Neutrophils in the Lungs of SA Mice

Besides lymphocytes, several myeloid (dendritic cells) and granulocytes such as macrophages, eosinophils, and neutrophils are critical effectors of immune response in asthma (10). Macrophages are the most abundant immune cells in the lung and critically regulate environmental allergen-induced airway inflammation (17, 18). Phenotypically, macrophages can be CD11c^+^ SiglecF^+^ MHCII^high^ (M1) or CD11c^+^ SiglecF^+^ MHCII^low^ (M2) sub-types that display inflammatory and anti-inflammatory functions, respectively (18). We observed that while the frequency of MHCII^low^ (M2) macrophages were greatly reduced in SA mice, there was an increase in the both the incidence and total numbers of MHCII^high^ (M1) cells in these animals (**Figures 4A** **–C**). Consistent with our data, prior studies have linked M1 macrophages with the pathogenesis of SA, particularly for those with a poor response to systemic CS (19). Next, we evaluated dendritic cells (DC) as they are principal antigen presenting cells and initiators of the immune response in allergic asthma (10). We observed a significant increase in the frequency and cell numbers of DC in SA mice (**Figures 4A** **–C**). Strikingly, while treatment with Ruxolitinib greatly reduced MHCII^high^ (M1) cells in SA+R mice, it had little-to-no effect on the cell counts of MHCII^low^ (M2) macrophages or DC (**Figure 4C**). Finally, we examined the effect of Ruxolitinib on the frequency and cell counts of eosinophils and neutrophils (**Figures 4D** **–I**), key effector cells in T2-high and T2-low asthma endotypes, respectively (2). Interestingly, we observed that the incidence and absolute numbers of both these cells were significantly increased in SA mice, reflecting a mixed granulocytic inflammation. Importantly, while treatment with Ruxolitinib had no effect on either the frequency or cell numbers of neutrophils (**Figures 4D** **–F**), it significantly reduced eosinophilc infiltration in the lungs of SA+R mice (**Figures 4G** **–I**).

### Ruxolitinib Targets Gene Expression of Several Chemokines but Not Alarmins in SA

**Figure 5:**
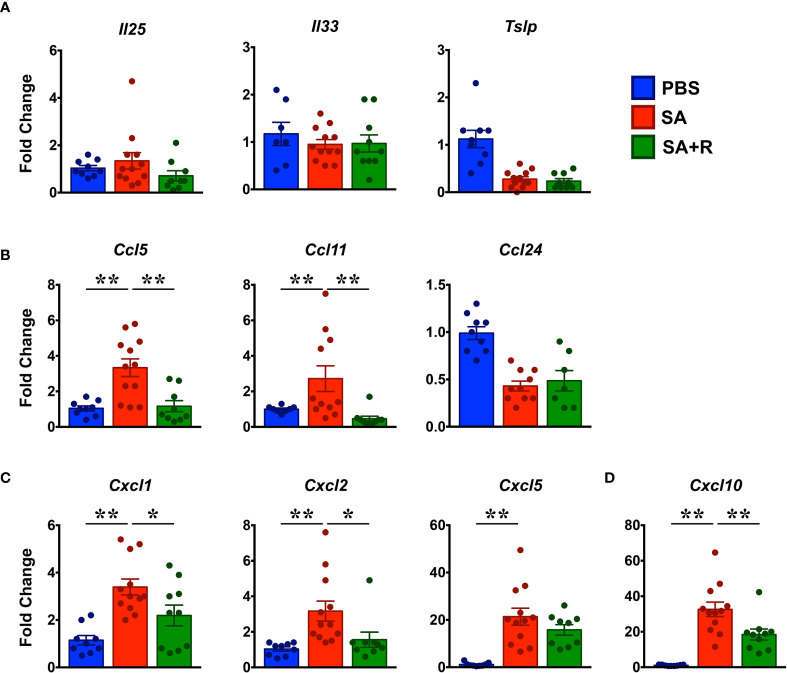
Reduced chemokine expression in the lungs of Ruxolitinib-treated SA mice. **(A–D)** Lungs tissues of PBS-treated, SA and SA+R mice were analyzed for gene expression of **(A)** alarmins (*Il25*, *Il33, Tslp*), and various eosinophilic **(B)**, neutrophilic **(C)**, and Th1 **(D)** chemokines by qPCR. Data is shown as mean fold change ± SEM and pooled from 3 independent experiments with a total of 8-12 mice per cohort. Statistical significance was determined by Student’s unpaired *t* test with Welch’s correction. *p ≤ 0.05, **p ≤ 0.01.

Alarmins such as thymic stromal lymphopoietin (TSLP), IL-25 and IL-33 can drive allergic inflammation in response to airway damage and their expression correlates with asthma severity (20). Surprisingly, we observed that gene expression of these alarmins in SA mice were either diminished (*Tslp*) or similar (*Il25, Il33*) to PBS controls. Moreover, their expression was unaltered by Ruxolitinib treatment (**Figure 5A**). Several chemokines, particularly CCL5, CCL11, and CCL24 are known to regulate eosinophil trafficking to the lungs (21, 22). Consistently, we observed that the mRNA levels of *Ccl5* and *Ccl11* were significantly upregulated in SA mice; however, gene expression of *Ccl24* was reduced in these animals (**Figure 5B**). Next, we examined chemokines that are known to promote the migration of neutrophils (CXCL1, CXCL2 and CXCL5) (22) and Th1 cells (CXCL10) (23) to the site of inflammation. We observed that gene transcripts of these chemokines were significantly upregulated in the lungs of SA mice (**Figures 5C, D**). However, while fold change (relative to PBS controls) in the gene expression of *Cxcl1* and *Cxcl2* ranged from 3.4 ±; 0.3 and 3.2 ±; 0.6 respectively; mRNA levels of *Cxcl5* (21.4 ±; 3.6) and *Cxcl10* (32.6 ±; 4.1) were substantially higher. Of note, with the exception of *Cxcl5*, Ruxolitinib significantly suppressed the expression of all the other chemokines that were elevated in SA mice (**Figures 5C, D**).

### Ruxolitinib Suppresses Th1 and T2 but Not Th17 Cytokines in SA Mice

**Figure 6:**
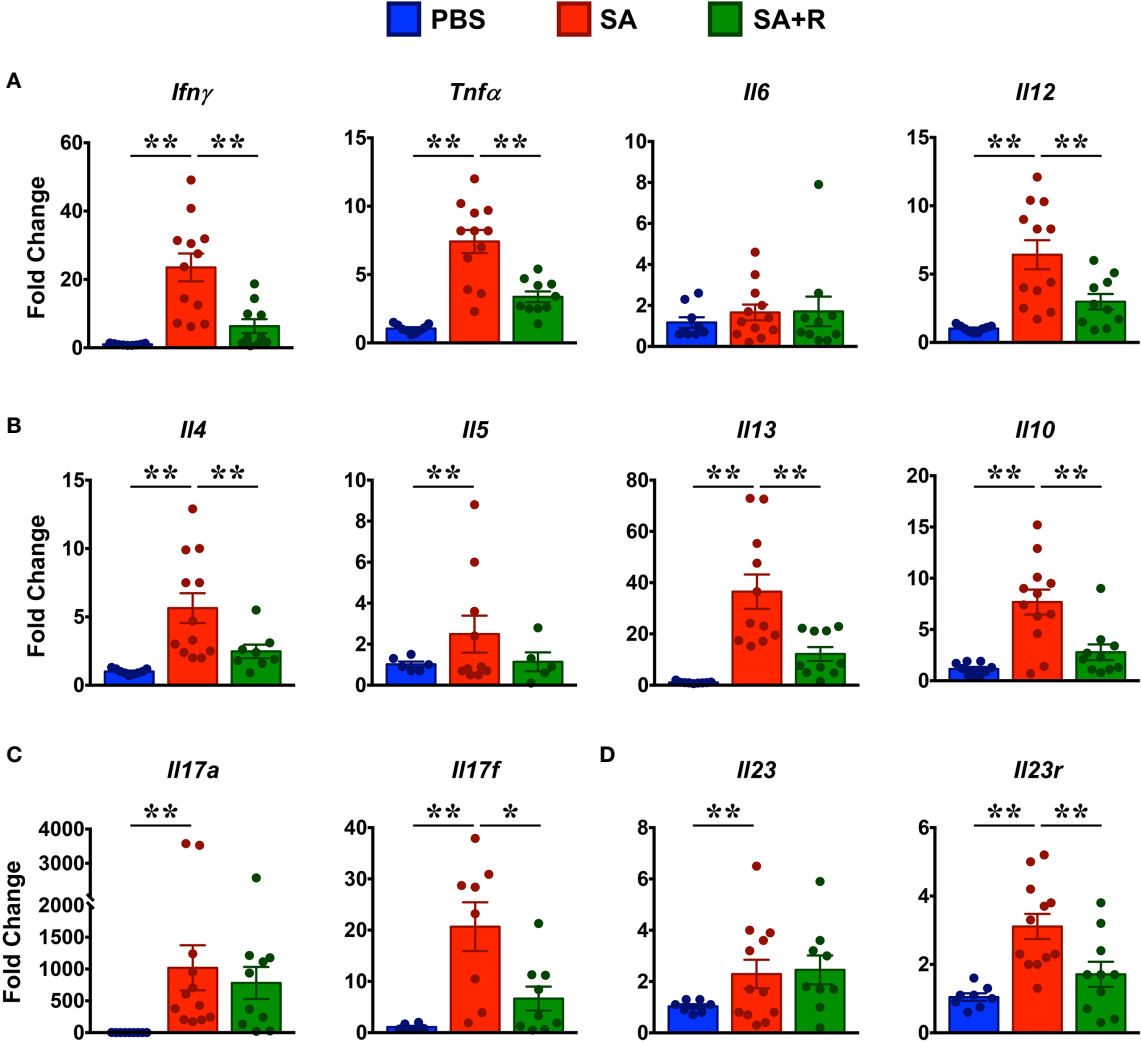
Ruxolitinib targets gene expression of several lung cytokines in SA mice. **(A–D)** Lungs of control, SA and SA+R mice were analyzed for gene expression of **(A)** Th1 (*Ifnγ*) and pro-inflammatory cytokines (*Tnfa, Il6, Il12)*, **(B)** T2 cytokines (*Il4, Il5, Il13* and *Il10*), **(C)** IL-17 family of cytokines (*Il17a* and *Il17f*),and **(D)** *Il23 *and* Il23r*. Data is presented as mean fold change ± SEM and is pooled from 3 independent experiments with a total of 8-12 mice per cohort. Statistical significance was determined by using Student’s unpaired *t* test with Welch’s correction. *p ≤ 0.05, **p ≤ 0.01.

CS-resistant severe asthmatics typically exhibit a T2-low asthma, wherein several pro-inflammatory cytokines including IFN-γ, TNF-α, IL-6 and IL-12 mediate its pathogenesis (1). So, we first assessed the gene expression of these cytokines and observed that with the exception of *Il6*, all the other pro-inflammatory cytokines were greatly increased in the lungs of SA mice (**Figure 6A**). Interestingly, gene expression of several T2 cytokines such as *Il4, Il5, Il1*3 and *Il10* were also upregulated in these animals (**Figure 6B**). Importantly, while gene expression of most of these cytokines were significantly reduced by Ruxolitinib, mRNA levels of *Il5* and *Il6* were comparable in SA and SA+R mice (**Figures 6A, B**). IL-17A and IL-17F are members of the IL-17 cytokine family and are produced by Th17 cells (24). In asthma patients, elevated levels of IL-17A and IL-17F are found in the airways and BALF that positively correlate with disease severity and neutrophil inflammation (1). Furthermore, IL-23 signaling is crucial for the maintenance and effector functions of Th17 cells (25). Consistent with these reports and increased neutrophilic infiltration (**Figures 4D** **–F**), we observed significantly elevated mRNA transcripts of *Il17a* and *Il17f* (**Figure 6C**) as well as *Il23* and *Il23r* (**Figure 6D**) in SA mice. However, while Ruxolitinib suppressed gene expression of *Il17f* and *Il-23r*, *Il17a* and *Il23* levels were comparable in SA and SA+R mice (**Figures 6C, D**).

**Figure 7:**
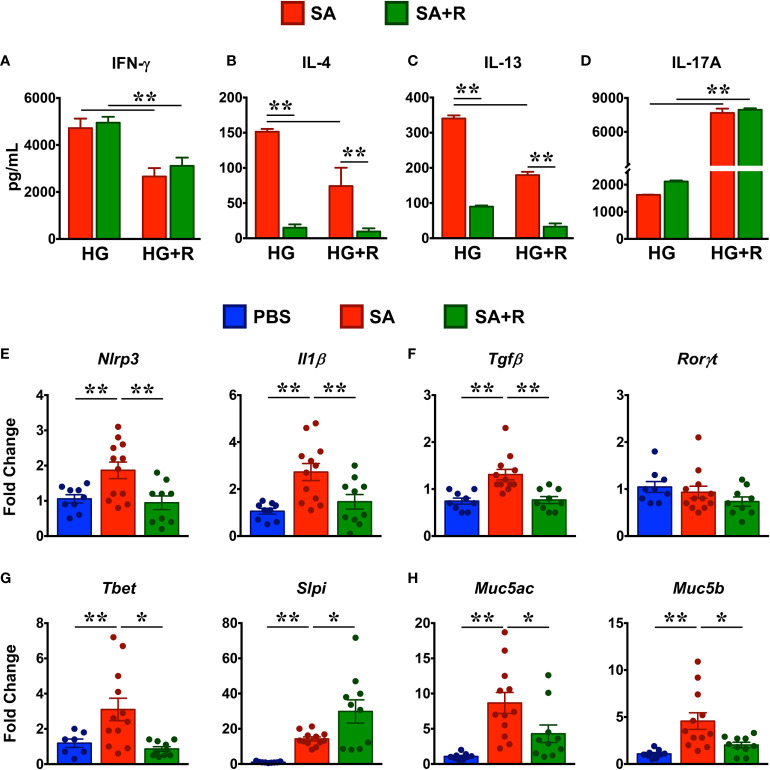
Treatment with Ruxolitinib suppresses Th1/Th2 cytokine production and associated gene expression in SA mice. **(A–D)** Lung immune cells from SA and SA+R mice were isolated and cultured *ex vivo* in the presence of either HMDE+c-di-GMP (HG) or HMDE+c-di-GMP+ Ruxolitinib (HG+R), as indicated. After 72 hours, cell culture supernatants were analyzed for IFN-γ **(A)**, IL-4 **(B)**, IL-13 **(C)**, and IL-17A **(D)**. Data is shown as mean ± SD and is from 1 representative experiment with n=3 mice per cohort. Statistical significance was determined by Student’s unpaired *t* test with Welch’s correction. **p ≤ 0.01. **(E–H)** Lung tissues from PBS-treated, SA and SA+R mice were analyzed for gene expression of **(E)** inflammasome pathway (*Nlrp3* and *Il1β*), **(F)** IL-17-associated genes (*Tgfβ* and *Rorγt*) **(G)** IFN-γ-regulating genes (*Tbet* and *Slpi*), and **(H)** polymeric mucins (*Muc5ac* and *Muc5b*). Data in **(E–H)** are presented as mean fold change ± SEM and are pooled from 3 independent experiments with a total of 9-12 mice per cohort. Statistical significance was determined using Student’s unpaired *t* test with Welch’s correction. *p ≤ 0.05 or **p ≤ 0.01.

To determine the effect of Ruxolitinib on the protein levels of Th1/Th2/Th17 cytokines, we cultured lung immune cells from SA and SA+R in the presence of HG without or with ruxolitinb (HG+R) *in vitro*. We observed that lung immune cells from both SA and SA+R mice produced robust levels of IFN-γ in the presence of HG that were significantly reduced in the presence of Ruxolitinib (**Figure 7A**). In contrast, SA mice produced modest amounts of IL-4 and IL-13 in the presence of HG and that were further suppressed in SA+R mice (**Figures 7B, C**). Furthermore, both these cytokines were significantly reduced when cells were stimulated *ex vivo* in the presence of Ruxolitinib (HG+R, **Figures 7B, C**). Strikingly, while HG-stimulated cells from SA and SA+R mice produced comparable IL-17A, these levels were significantly increased when cells were cultured in presence of HG+R (**Figure 7D**).

### Ruxolitinib-Treated SA Mice Exhibit Reduced Expression of NLRP3, IL-1β and Mucins

Excessive nucleotide-binding oligomerization domain-like receptor family, pyrin domain-containing 3 (NLRP3) inflammasome and concomitant IL-1β responses play a critical role in steroid-resistant SA (26) and promote Th17 allergic asthma in mice (27). Given that SA+R mice had elevated levels of IL-17A, we determined the effect of ruxoltinib on the gene expression of NLRP3 and IL-1β. Consistent with the well-documented role for the NLRP3-IL-1β axis in the pathogensis of SA (28), we observed increased gene transcripts of these molecules in the lungs of mice challenged with HG (**Figure 7E**). In conjunction with IL-1β, TGF-β also promotes Th17 development (25). Accordingly, gene expression of *Tgfβ* was also increased in SA. Importantly, mRNA expression *Nlrp3*, *Il1β* and *Tgfβ* were significantly reduced in SA+R mice (**Figures 7E, F**), suggesting that the persistent high IL-17A levels in these mice was independent of the NLRP3-IL-1β axis as well as TGF-β. Furthermore, gene expression of *Rorγt* (lineage-defining transcription factor of Th17 cells) (25) was neither elevated in SA nor altered in SA+ R mice, as compared to levels in PBS controls (**Figure 7F**).

T-bet is a critical regulator of Th1 lineage commitment and IFN-γ production (29). In contrast, secretory leukocyte protease inhibitor (SLPI) negatively regulates IFN-γ3. However, we observed that the mRNA levels of both *Tbet* and *Slpi* were increased in SA mice relative to PBS controls. Interestingly, while mRNA expression of *Tbet* was suppressed, *Slpi* gene transcript was significantly increased in SA+R mice (**Figure 7G**).

The direct downstream target of IL-13 and to some extent of IL-4, is mucus production by goblet cells in the airways (30). Goblet cell hyperplasia partly contributes to airway remodeling by mucus hypersecretion and airflow obstruction (31). In mice, excess mucus production is regulated by 2 polymeric mucins: Muc5ac and Muc5b (31). We observed that both *Muc5ac* and *Muc5b* gene transcripts were significantly upregulated in the lungs of SA mice. Importantly, in agreement with reduced IL-4 and IL-13 levels in SA+R mice, mRNA levels of both *Muc5ac* and *Muc5b* were markedly reduced by treatment with Ruxolitinib (**Figure 7H**).

### Ruxolitinib Regulates miRNA Expression in the Lungs of SA Mice

**Figure 8:**
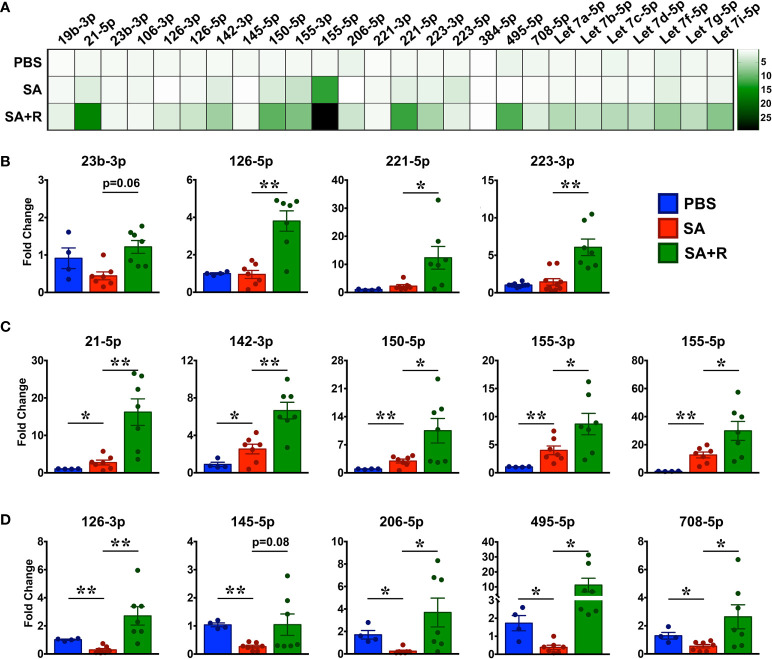
Increased expression of various miRNAs in the lungs of SA mice treated with Ruxolitinib. Lungs tissues of PBS-injected, SA and SA+R mice were analyzed for gene expression of various miRNAs as indicated in the figure. **(A)** Heat map of gene expression of various miRNAs in the lungs of SA and SA+R mice, normalized to PBS controls. **(B–D)** Fold change in expression of various miRNAs in the lungs of SA and SA+R mice with known roles in asthma pathogenesis. Fold change for each cohort was normalized to PBS-treated mice. Data is presented as mean ± SEM and are pooled from 2 independent experiments with a total of n=4 mice for PBS controls and n=7 mice for SA and SA+R groups. Statistical significance was determined using Student’s unpaired *t* test with Welch’s correction. *p ≤ 0.05 or **p ≤ 0.01.

Several microRNAs (miRNAs) play either a protective or proinflammatory role in asthma (32). As depicted in the heat map, we screened for various miRNAs that have known roles in different aspects of asthma pathogenesis (**Figure 8A**). We observed that while PBS controls and SA mice had comparable levels of several miRNAs including miR23b-3p, miR126-5p, miR221-5p and, miR223-3p (**Figure 8B**), expression of miR21-5p, miR142-3p, miR150-5p, miR155-3p and miR155-5p were significantly increased in SA mice (**Figures 8C**). In contrast, gene transcripts of miR126-3p, miR145-5p, miR206-5p, miR495-5p and miR708-5p were greatly reduced in SA mice as compared to the PBS controls (**Figures 8D**). Importantly, expression of these miRNAs were significantly increased in SA+R mice (**Figures 8B** **–D**). Additionally, we observed an upward trend in the expression of most Let-7 miRNAs in these mice (**Figure 8A**). As several of these miRNAs have established roles in airway smooth muscle cell (ASMC) proliferation, AHR and lung inflammation, our data suggest that treatment with Ruxolitinib suppresses the pathogenesis of SA, at least in part *via* regulation of miRNAs.

## Discussion

Traditional therapy for asthma includes inhaled or systemic CS, bronchodilators, and anti-leukotrienes (1, 33). However, asthma is a complex heterogeneous disease with varying phenotypes and endotypes (1, 2, 33). As such, traditional therapies do not cater to all the asthma patients. Furthermore, prolonged use of CS may lead to several adverse effects including heart disease, infections, osteoporosis, bone fracture, stroke, and cataract (2). Importantly, 5-10% patients are refractory to mainstay asthma treatments and contribute significantly to the morbidity and mortality associated with asthma (33). These subjects have SA and exhibit a high Th1/IFN-γ immune response in spite of ongoing CS treatment (1). The emergence of new biologics offers alternative options to improve asthma control. While some of these targeted therapies seem promising, others have potential limitations (2, 20). For example, FDA-approved anti-IgE monoclonal antibody (mAb), is applicable for a subset of patients that have uncontrolled IgE-mediated allergic asthma. Other biologics include mAbs against IL-5, IL-4 and IL-13 and their respective cognate receptors. However, these mAbs primarily target immune cells and cytokines implicated in the T2-high inflammation. Several cytokines are pivotal in the regulation of airway inflammation and tissue remodeling by their actions on immune, epithelial and ASMCs (33). Thus, targeting a single cytokine or inflammatory cell may not be sufficient to reduce inflammation, possibly because of compensatory response from a different cytokine. Therefore, developing drugs that can suppress multiple cytokines may be a more effective strategy.

As most pro-inflammatory cytokines signal through the JAK-STAT pathway (4), JAK inhibition is a potential therapeutic target for the treatment of asthma (5, 6). Indeed, prior studies have shown that several oral JAK inhibitors can reduce airway and lung inflammation in ovalbumin (OVA)-induced murine model of asthma (34–36). However, prolonged use of oral JAK inhibitors increase the risk of systemic side effects such as lymphopenia, neutropenia, infections, intestinal damage, liver dysfunction as well as malignancies (37). Moreover, the effect of JAK inhibition on CS-resistant SA remained to be evaluated. To directly address these issues, in the current study, we determined the effect of inhaled JAK inhibition on the immunopathology of CS-resistant SA.

Our studies reveal several important observations. First, Ruxolitinib reduces AHR by downregulating IFN-γ but not IL-17A cytokine levels. Indeed, previously it has been shown that IFN-γ but not IL-17A promote AHR (3). In the same study, the authors demonstrate an inverse correlation between IFN-γ and SLPI and that increased SLPI levels attenuate AHR (3). However, it is worth noting that SA patients exhibit elevated levels of SLPI, albeit at significantly lower extent than those observed in mild-moderate asthmatics (3). Consistently, we observed that although *Slpi* is significantly increased in SA, its expression is considerably lower when compared to SA+R mice. Interestingly, SLPI negatively regulates the activation of TGF-β (38), a key mediator of airway remodeling during asthma progression (39). It is well established that TGF-β promotes aberrant proliferation and proinflammatory effects of ASMC, the principal effectors of AHR (40, 41). Additionally, TNF-α and IL-13 can also promote AHR by either directly acting on ASMC or by inducing the release of various inflammatory mediators (42) or by augmenting mucus secretion that results in thickening of the airways (30). As the expression of *Tgfβ*, *Tnfα* and *Il13* as well as its downstream targets, *Muc5ac* and *Muc5b* were dampened by Ruxolitinib, our data indicate that JAK1/2 inhibition ameliorates AHR by affecting multiple pathways.

Second, Ruxolitinib alters the lung immune landscape and inhibits the expression of several mediators that can amplify and/or perpetuate the Th1 inflammation associated with CS-resistant SA. To that end, CD8+T, iNKTs, DCs and M1 macrophages were elevated in SA mice. This was accompanied by increased expression of *Tbet*, several proinflammatory cytokines (*Ifng, Il12, Tnfa*, and *Il1β*), and the chemokine *Cxcl10*. T-bet is not only a potent inducer of IFN-γ (43) but is also critical for Th1/Th2 differentiation of CD4+ T cells (43, 44). It is also expressed by antigen-presenting cells (APCs), including DCs for optimal production of IFN-γ and subsequent antigen-specific Th1 cell activation (45, 46). It is interesting to note that while T-bet was increased in SA mice, CD8+ T cells were not as numerous as CD4+ T cells in the lungs of these animals. However, as both CD4+ and CD8+ T cells were increased in SA mice, our data highlight a co-operative role for these T cell subsets in AHR and airway inflammation. Indeed, prior studies have shown that CD4+ T cells are required for CD8+ T cell activation (47) and when co-transferred at the same time, they drive the most severe airway response (48).

IL-12 is produced by activated monocytes, macrophages, and DCs that direct the differentiation of T cells into Th1 cytokine-producing cells while suppressing Th2 polarization (10). Additionally, IFN-γ and IL-12 promote M1 macrophage differentiation that in turn produce more Th1 cytokines including TNF-α, IL-1β, IL-6, and IL-12, establishing a positive feedback loop (17, 18). Furthermore, IL-12 augments NK and CD8+ T cell proliferation as well as IFN-γ production by these cells (49). TNF-α is a pleiotropic cytokine that recruits various inflammatory cells into the lung tissue and activates them to produce several proinflammatory cytokines (including TNF-α itself) and chemokines (50). Notably, TNF-α and IFN-γ individually or synergistically can enhance CXCL10 production, a strong chemoattractant for Th1 cells (50). Consistently, CXCL10 is elevated in the BAL cells of CS-resistant SA patients (23), as observed in our experimental mice. Except for IL-6, Ruxolitinib significantly reduced the expression of all these inflammatory mediators. Accordingly, the immune populations (CD8+ T, NK and M1 macrophage) producing these cytokines and/or being affected by them, were dramatically fewer in the lungs of SA+R mice. In contrast, DC numbers were not affected by Ruxolitinib. As DC are the major source of IL-12 and TNF-α10, our studies indicate that JAK1/2 inhibition primarily impairs DC functions. Indeed, a prior study has shown that Ruxolitinib affects DC functions leading to impaired T cell activation (51).

Third, Ruxolitinib suppresses the T2 immune response associated with CS-resistant SA. This is evident by reduced lung eosinophilia and IgE production in SA+R mice. Elevated IgE levels in the serum is a hallmark feature of allergic asthma (10), where T2-cytokines IL-4 and IL-13 promote B cell differentiation, isotype class switching and IgE secretion (10). Moreover, in the presence of IL-4, IL-10 a major regulatory cytokine enhances B cell proliferation and IgE synthesis (52). Thus, the reduced levels of serum IgE in SA+R mice can be attributed to the diminished expression of these T2 cytokines as well as to the significantly fewer B cell numbers in lungs of these mice. Airway eosinophilia is not only characteristic of T2-high asthma but is also present in T2-low asthma (1). Indeed, steroid-insensitive patients with eosinophil-enriched inflammation have been described (53), consistent with our data. IL-5 is a key cytokine for the differentiation, activation and survival of eosinophils and together with IL-4 and IL-13, it regulates the recruitment of these cells into the lungs (54). As *Il5* mRNA levels were unaffected by Ruxolitinib, yet eosinophilia was greatly reduced in SA+R mice, our observations suggest that both IL-4 and IL-13 drive eosinophilic inflammation in the lung. However, IL-13 has been touted as the key mediator of T2-dominant immune responses (30, 54, 55), perhaps due to its greater production and persistence (56). Indeed, we observed that IL-13 was much higher than IL-4 in the lung tissue and culture supernatants from SA mice, akin to data from CS-resistant SA patients (3).

Several chemokines, particularly CCL5, CCL11, and CCL24 are also involved in the airway recruitment of eosinophils (22). Strikingly, we observed that *Ccl5* and *Ccl11* but not *Ccl24* levels were increased in SA mice. CCL5 and CCL11 are both highly expressed by airway epithelial cells, however, CCL5 is produced by Th1 and CCL11 by Th2 cells (22). Importantly, CCL5 can induce receptor-mediated calcium flux and chemotaxis while CCL11 primarily mediates eosinophil degranulation (22). Additional studies have shown that CCL5 plays a role in the regulation of airway function through modulation of IFN-γ and IL-12 levels (57) and that both these chemokines contribute to the irreversible airflow obstruction in SA (58). Given that both *Ccl5* and *Ccl11* were dramatically reduced in SA+R mice, our studies reveal that JAK1/2 inhibition can ameliorate lung inflammation not only by targeting eosinophil chemotaxis and function but also by interfering with the cross-regulation of the Th1 and T2 inflammation that co-exist in steroid-insensitive patients.

Fourth, Ruxolitinib impairs the NLRP3-IL-1β axis but fails to resolve the neutrophilic infiltration observed in SA mice. Activation of NLRP3 inflammasome leading to IL-1β secretion contributes to lung inflammation *via* Th17 differentiation and IL-17 production (26, 28). Consistent with this notion, we observed increased expression of *Nlrp3, Il1β* as well as *Il17a and Il17f* in SA mice. However, while *Nlrp3, Il1β *and* Il17f* levels were significantly reduced in SA+R mice, *Il17a* remained unaltered, suggesting that either NLRP3 does not regulate IL-17A production in our model or that there are additional mechanisms at play. Alternatively, it is also possible that NLPR3 contributes to the airway inflammation independent of IL-17A. Indeed, NLRP3-deficient mice exhibit dramatically reduced eosinophil recruitment, mucus secretion and IL-1β that correlate with reduced Th2 cytokine and chemokine production (59). Several cytokines including IL-6, TGF-β, IL-1β and IL-23 promote differentiation of CD4+T cells to Th17 cells (25). Another source of IL-17A in the lungs is iNKT cells (15). As both CD4+ T and iNKT cell numbers were preserved in SA+R mice, these cells are likely to contribute to the sustained IL-17A levels in our model. Surprisingly, we observed very little *Il6* in the lungs of SA mice, however, it is possible that IL-6 is elevated during the sensitization phase and reduces to baseline levels in the challenge phase. Whether these or other mechanisms regulate the IL-6-IL-17 axis in the SA mice remain to be evaluated.

IL-17 plays a critical role in neutrophil recruitment to the lungs by regulating expression of several chemokines, including CXCL1, CXCL2 and CXCL5 (28); all of which were elevated in SA mice. Interestingly, expression of *Cxcl1* and *Cxcl2* was decreased by Ruxolitinib, yet neither the frequency nor the number of neutrophils were reduced in SA+R mice. Notably, like *Il17a*, *Cxcl5* was unaffected by Ruxolitinib and its expression was much higher than *Cxcl1* and *Cxcl2* indicating that lung neutrophilia observed in the SA+R mice is mediated by the IL-17A-CXCL5 axis. Functionally, neutrophils can be proinflammatory or immunosuppressive and can exert adverse or beneficial effects in asthma (60). Given the considerable attenuation of disease severity in our model, we favor a protective role for these cells. Indeed, a protective role for neutrophils in limiting HDME- induced allergic airway inflammation has been reported (61). Contrary to our observations, a recent study demonstrated that Ruxolitinib can reduce neutrophilic inflammation (62). This discrepancy is attributable to the differences in the allergens used as well as the route, dose and timing of Ruxolitinib administration. Indeed, we demonstrate that Ruxolitinib can significantly decrease or increase IL-17A levels in culture supernatants from lung cells, depending on whether the cells were stimulated after the sensitization (**Figure 1D**) or the challenge phase (**Figure 7D**), respectively.

Finally, we demonstrate that Ruxolitinib upregulates the expression of several miRNAs linked to multiple aspects of asthma pathogenesis. We observed that of the few miRNAs that were elevated in the lung tissues of SA mice, miR155-5p was most highly upregulated. Indeed, studies have shown that miR155 overexpression promotes Th1 responses upon IFN-γ stimulation (63), induces a pro-inflammatory M1 macrophage response, and enhances IL-12 production by DC (64). Additionally, it can directly target the transcription factor PU.1. to promote Th2 allergic inflammation (65). Surprisingly, of the many miRNAs that were upregulated in the SA+R mice, miR155-5p was among the most highly expressed, even beyond levels seen in SA mice. As Ruxolitinib significantly ameliorated both T1 and T2 immune response, it is reasonable to speculate that higher miR155-5p expression in SA+R confers protection. Consistent with our hypothesis, studies have shown that low miR155 expression favors IL-1β production, however high miR155 levels suppress gene expression of IL-1 and other proinflammatory cytokines (66). Importantly, miR155 inhibits IFN-γ signaling in CD4+ T cells (67) and also downregulates T2 responses by directly targeting the transcription factor, c-Maf (68) and IL-13 receptor α1 (69).

Increased expression of several miRNAs inhibit ASMC proliferation or AHR *via* different pathways. Specifically, miR23b-3p (70) and miR142-3p (71) inhibit TGFβ-dependent ASMC proliferation while miR150-3p and miR708-5p inhibit ASMC proliferation by downregulating BCYRN1 (72) and CD38 (73), respectively. Moreover, miR145-5p alleviates airway remodeling by targeting EGFR to downregulate Muc5ac expression (74). On the other hand, several miRNAs promote T2 effector functions. For example, miR-21 potentiates Th2 polarization by suppressing Th1 differentiation (75, 76), miR126 promotes eosinophil recruitment (77) and miR206 upregulates airway IL-25 and TSLP levels (78). In contrast, Let-7 miRNAs, miR150 and miR495 downregulate T2 responses by targeting IL-13 (79) or the transcription factor, GATA-3 (80, 81). Additionally, while miR221 promotes Th1 inflammation (64), miR223 inhibits the NLRP3/IL-1β axis (82) as well as granulocyte progenitor cell differentiation and activation (83). Strikingly, all these miRNAs were increased in SA+R mice. Collectively, our data underscore the complexity of the miRNA regulatory networks that co-operate with or cross regulate each other to ameliorate AHR and lung inflammation. However, the exact mechanism by which Ruxolitinib impacts expression of these miRNAs in various immune and non-immune cells, warrants further investigation.

In conclusion, we demonstrate that intranasal administration of low dose Ruxolitinib dramatically reduces AHR, lung inflammation and IgE production in a mouse model of SA. This amelioration is linked to the suppression of several chemokines, cytokines, and downstream inflammatory mediators that drive asthma pathogenesis in steroid-unresponsive patients. Ruxolitinib is a potent, highly bioavailable JAK1/2 inhibitor that is already US Food and Drug Administration (FDA) approved for the treatment of myelofibrosis, polycythemia vera, steroid-refractory acute graft versus host diseases and atopic dermatitis (84). Importantly, Ruxolitinib targets inflammation differently than steroids, and thus, it can be used independently or in combination with steroids to treat severe asthmatics more effectively. Recently, two reports have shown that local inhibition of JAK in the lung suppresses OVA-induced lung inflammation and AHR (85, 86). Together with our data, these studies provide evidence that JAK inhibition by the inhaled route provides a promising alternative for both mild and severe asthma treatment.

## Data Availability Statement

The original contributions presented in the study are included in the article/Supplementary Material. Further inquiries can be directed to the corresponding author.

## Ethics Statement

The animal study was reviewed and approved by Institutional Animal Care and Use Committee at the Michigan State University.

## Author Contributions

TH performed experiments and analyzed data. AK performed experiments and assisted with AHR data analysis. DB performed FACS staining, ELISA and qPCR for miRNA analysis. KT performed ELISA and assisted in organ processing for qPCR and histology. HS and RD designed and supervised the research, analyzed the data and wrote the manuscript. All authors read and edited the article and approved the submitted version.

## Funding

This work was supported by grants from the National Institutes of Health to RD (5K22CA18814802) and HS (5R00HL12107305). KT was a NIH-NHLBI scholar, her research training was supported through an NIH 5-R25-HL108864-award to Dr. Elahě Crockett, Director of the Research Education Program to Increase Diversity in Health Researchers (REPID), Michigan State University.

## Conflict of Interest

The authors declare that the research was conducted in the absence of any commercial or financial relationships that could be construed as a potential conflict of interest.

## Publisher’s Note

All claims expressed in this article are solely those of the authors and do not necessarily represent those of their affiliated organizations, or those of the publisher, the editors and the reviewers. Any product that may be evaluated in this article, or claim that may be made by its manufacturer, is not guaranteed or endorsed by the publisher.
